# Developmental programmed cell death during asymmetric microsporogenesis in holocentric species of *Rhynchospora* (Cyperaceae)

**DOI:** 10.1093/jxb/erw300

**Published:** 2016-08-04

**Authors:** Danilo M. Rocha, André Marques, Celia G.T.J. Andrade, Romain Guyot, Srinivasa R. Chaluvadi, Andrea Pedrosa-Harand, Andreas Houben, Jeffrey L. Bennetzen, André L.L. Vanzela

**Affiliations:** ^1^Laboratory of Cytogenetics and Plant Diversity, Department of General Biology, Center of Biological Sciences, State University of Londrina, Londrina 86057-970, Paraná, Brazil; ^2^Laboratory of Plant Cytogenetics and Evolution, Department of Botany, Federal University of Pernambuco, Recife, Brazil; ^3^Laboratory of Electron Microscopy and Microanalysis, Pro-PPG, State University of Londrina, 86051990, Londrina, Brazil; ^4^Institut de Recherche pour le Développement (IRD), UMR IPME, BP 64501, 34394, Montpellier Cedex, France; ^5^Department of Genetics, University of Georgia, Athens, GA 30602, USA; ^6^Leibniz Institute of Plant Genetics and Crop Plant Research (IPK) Gatersleben, D-06466 Stadt Seeland, Germany

**Keywords:** Asymmetric cell plate, electron microscopy, holocentromeres, meiotic drive, mitotic disorder, pollen development, vacuolar cell death.

## Abstract

Members of the Cyperaceae family exhibit an asymmetric microsporogenesis that results in the degeneration of three out of four meiotic products. Efforts have been made previously to describe the resulting structure, named the pseudomonad, but mechanisms concerning the establishment of cell domains, nuclear development, and programmed cell death are largely unknown. Using the *Rhynchospora* genus as a model, evidence for cell asymmetry, cytoplasmic isolation, and programmed cell death was obtained by a combination of electron microscopic, cytochemical, immunocytochemical, *in situ* hybridization, and flow cytometric methods. Degenerative cells were identified at the abaxial region, with the cytoskeleton marking their delimitation from the functional domain after meiosis. After attempting to initiate cell division with an unreplicated genome and abnormal spindle assembly, these cells exhibited a gradual process of cytoplasmic contraction associated with hypermethylation of cytosines and differential loss of DNA. These results indicate that the asymmetric tetrad establishes a functional cell, where one nucleus is preferentially selected to survive. Degenerative haploid cells are then eliminated in a multistep process associated with mitotic disorder, non-random elimination of repetitive DNA, vacuolar cell death, and DNA fragmentation.

## Highlight

The functional cell of an asymmetric tetrad is selected according to its replication capability, while degenerative cells progress to multistep PCD, including mitotic disorder and DNA elimination.

## Introduction

The sedge family Cyperaceae is the third largest of monocotyledonous plants (after grasses and orchids), containing ~5000 species (Goetghebeur, 1998), of which many are important components of wetland ecosystems worldwide. Its members are distinctive due to holocentric chromosomes (Vanzela and Guerra, 2000; Melters *et al.*, 2012). Chromosomes of *Rhynchospora* contain satellite repeat sequences named Tyba, intercalated with CENPH3 nucleosomes, that are potential attachment sites to the kinetochore, as observed in *Rhynchospora pubera* (Marques *et al.*, 2015). Sedges also exhibit an asymmetric microsporogenesis (Furness and Rudall, 1999; Ranganath and Nagashree, 2000; Simpson *et al.*, 2003; San Martin *et al.*, 2013). After meiosis, the tetrad exists as four nuclei in a coenocytic wedge-shaped cell (Simpson *et al.*, 2003). One nucleus occupies the center of the cell, and the other three nuclei can be located either in the narrower apex placed near the anther axis, referred to here as the adaxial pole, or at the broader apex adjacent to the tapetum tissue, named the abaxial pole. This structure is then asymmetrically separated by cell wall formation, creating one functional domain containing one cell and one degenerative domain with three cells (Brown and Lemmon, 2000; San Martin *et al.*, 2013). The resulting structure is called a pseudomonad (Håkansson, 1954).

The functional cell of a pseudomonad undergoes an asymmetric mitosis, known as pollen mitosis I (PM I), to give rise to the generative and vegetative cells, as typical for most angiosperms (Furness and Rudall, 1999; Brown and Lemmon, 2000). The degenerative cells may initiate a division, but in almost all cases they are soon arrested (Maheshwari, 1950; Ranganath and Nagashree, 2000). During pseudomonad development, the degenerative cells become successively smaller, until they are completely aborted by programmed cell death (PCD) (Kirpes *et al.*, 1996; Ranagath and Nagashree, 2000; Coan *et al.*, 2010; San Martin *et al.*, 2013). The pseudomonad was considered a synapomorphy of the Cyperaceae (Kirpes *et al.*, 1996; Coan *et al.*, 2010), although there are doubts about the pollen development process in some basal species (Simpson *et al.*, 2003). Pseudomonads are not exclusive to the Cyperaceae, as they have also been described in members of Epacridaceae R. Br. (Furness and Rudall, 2011).

Although pseudomonads in Cyperaceae represent an exciting model for studies of cell asymmetry and nuclear selection associated with PCD, the mechanism behind this cellular organization, as well as its evolutionary meaning, remain unknown. The positioning of degenerative cells in the apex of pseudomonads and its compartmentalization into small cytoplasmic environments has led to the assumption that degeneration is an outcome of their distant confinement from the tapetum (Håkansson, 1954; Strandhede, 1973; Kirpes *et al.*, 1996). However, pseudomonads in *Rhynchospora* species present degenerative nuclei in their abaxial region, next to the tapetum (Tanaka, 1941; Coan *et al.*, 2010; San Martin *et al.*, 2013). Asymmetry can also be observed in PM I, which generates two unequal cells, one of them being the small generative cell, which, although similarly confined in a small cytoplasmic space, is fully functional.

Furness and Rudall (2011) have suggested that the polarized tetrads observed in Cyperaceae male meiosis might be correlated with meiotic drive, probably because distortions in the Mendelian segregation associated with unsuccessful gamete production may be related to asymmetric tetrad development. Meiotic drive is usually expected to occur during meiosis of female animals and megasporogenesis in seed plants, wherein all but one of the meiotic products usually degenerate (Fishman and Saunders, 2008). The potential relationship between pseudomonad development and meiotic drive needs to be further investigated. There have been efforts to characterize the formation of pseudomonads (Håkansson, 1954; Brown and Lemmon, 2000; Coan *et al.*, 2010; Furness and Rundall, 2011; San Martin *et al.*, 2013). However, there are still many gaps in our knowledge regarding the delimitation and selection of cytoplasmic environments (functional and degenerative), as well as the molecular pathways and the evolutionary meaning of nuclear degeneration.

In this study, we investigate how pseudomonads are established using the genus *Rhynchospora* (Cyperaceae) as a model. We used a combination of tools to describe pseudomonad formation in sister species *R. breviuscula* and *R. pubera*. Several main questions guided this work. (i) Could the cell plate ensure the stability of both functional and degenerative cytoplasm? (ii) What would happen if asymmetric cell plate formation was disrupted? (iii) Do degenerative nuclei appear smaller than the functional nucleus in pseudomonads due to loss of repetitive DNA sequences, DNA under-replication, or chromatin hypercondensation? (iv) What kind of PCD predominantly occurs in degenerative cells? Herein we provide insights into all of these questions.

## Materials and methods

### Plant material

Ten individuals each of *R. breviuscula* and *R. pubera* were collected in Iporanga, São Paulo, Brazil and Curado, Recife, Brazil, respectively. Plants were kept in a greenhouse of the Laboratory of Cytogenetics and Plant Diversity (LCDV) at the State University of Londrina, Brazil and in the Experimental Garden of the Laboratory of Plant Cytogenetics and Evolution of the Federal University of Pernambuco, Brazil. Vouchers were deposited in the FUEL herbarium.

### Light microscopic analysis

Anthers were processed by several different protocols for microscopy. (i) Samples were fixed in a modified Karnovsky’s solution (Karnovsky, 1965) composed of 2.5% glutaraldehyde and 2.5% paraformaldehyde in 0.1M sodium cacodylate buffer (pH 7.2) and post-fixed in 1% osmium tetroxide. The material was dehydrated in a graded ethanol series, processed through propylene oxide, and embedded in Araldite resin^®^. Semi-thin sections were stained with 2% Toluidine blue O in sodium borate buffer. (ii) Anthers were fixed in absolute ethanol:glacial acetic acid (3:1, v/v) and kept at −20 °C until use. Samples were washed in distilled water, treated with 50% Ag(NO)_3_ for 12h at 60 °C, and dissected in a drop of 45% acetic acid. Coverslips were removed after freezing in liquid nitrogen and slides were mounted using Entellan (Merck). (iii) Anthers were directly dissected in 1% Alexander stain (Alexander, 1969) and prepared as semi-permanent slides. Images were acquired using a Leica DM4500B microscope equipped with a Leica DFC300FX camera.

### Cytochemical and immunocytochemical detection of cytoskeletal elements and histone modification

Cytoskeletal elements were studied using three different procedures. For cytochemical detection of F-actin, anthers were fixed in 4% paraformaldehyde in 1× phosphate-buffered saline (PBS) for 1h, washed in 1× PBS, and digested in an enzymatic solution containing 2% cellulase, 20% pectinase, and 2% lyticase (v/w/v). Materials were dissected in 1× PBS, and coverslips were removed after freezing in liquid nitrogen. Slides were treated with 5% phalloidin–fluorescein isothiocyanate (FITC), which binds strongly only to actin microfilaments, and later stained for DNA using 2 µg ml^–1^ DAPI solution. Afterwards, slides were mounted using antifade solution (25 µl) composed of DABCO [1,4-diaza-bicyclo(2.2.2)-octane (2.3%)], 20mM Tris–HCl pH 8 (2%), with 2.5 mmol l^–1^ MgCl_2_ (4%), and glycerol (90%), in distilled water.

Immunolabeling of the pseudomonads using anti-alpha-tubulin and anti-CENH3 antibodies was performed as described in Cabral *et al.* (2014), with modifications. First, anthers were fixed in methanol:acetic acid (3:1, v/v) for 24h. Afterwards, the anthers were dissected in 1× PBS. Coverslips were removed after freezing in liquid nitrogen, and the slides were immediately washed in 1× PBS. RpCENH3 antibody (Marques *et al.*, 2015) was detected with Cy3–goat anti-rabbit antibody. Mouse anti-α-tubulin antibody (Sigma, #T5168) was diluted 1:50 in 1× PBS containing 1% BSA and detected with Alexa 488–goat anti-mouse antibody (ThermoFisher, #A-11001) diluted 1:100 in the same buffer.

For disrupting microtubules, fresh inflorescences were treated with a drop of a 0.5% agarose gel containing 0.05% colchicine for 48h at room temperature and protected from light. For each treatment, a control was made in the same conditions without colchicine. Anthers were collected and fixed in absolute ethanol:glacial acetic acid (3:1, v/v) and stored at −20 °C until use. Samples were digested with the same enzymatic solution previously described and dissected in a drop of 45% acetic acid. After coverslip removal in liquid nitrogen, slides were stained with 2 µg ml^–1^ DAPI and mounted in DABCO, as described above. Widefield fluorescence images were recorded using a Leica DM5500B microscope equipped with a Leica DFC FX camera and a deconvolution system.

### Search and localization of repetitive DNA families

A genomic DNA library of *R. breviuscula* was produced using the CopyControl™ HTP Fosmid Library Production Kit (Epicenter, USA) according to the manufacturer’s instructions. A small portion of the library of *R. breviuscula*, consisting of 45 randomly selected fosmids (with ~35–50kb inserts) was sequenced as a pool using Illumina MiSeq technology. Sequences were processed with the FASTx-toolkit (http://hannonlab.cshl.edu/fastx_toolkit/commandline.html) that produced an output file containing 2 582 108 short reads (30–101bp). Repetitive sequences were assembled with the AAARF tool (DeBarry *et al.*, 2008), which produced 3908 contiguous sequences ranging from 86bp to 33 246bp in length. The assembled repeats were compared with repetitive elements in RepBase at http://www.girinst.org/censor (Jurka *et al.*, 2005), Gypsy Database 2.0 at http://gydb.org/index.php/Blast (Llorens *et al.*, 2011), and the NCBI databases (https://www.ncbi.nlm.nih.gov/). These same raw reads and assemblies were used to check for the presence of typical satellite DNA motifs using the RepFind (http://cagt.bu.edu/page/REPFIND_submit) and Tandem Repeats Finder (http://tandem.bu.edu/trf/trf.html) tools. Primers for PCR amplification of these repeats were designed using Primer3 (http://www.bioinformatics.nl/cgi-bin/primer3plus/primer3plus.cgi/).

For fluorescent *in situ* hybridization (FISH), slides were prepared by dissection in a drop of 45% acetic acid of anthers previously fixed in ethanol:glacial acetic acid (3:1, v/v) and digested in enzymatic solution as previously described. Coverslips were removed after freezing in liquid nitrogen. Sequences were obtained by PCR using specific primers for the centromeric-specific repeat Tyba-satDNA (F 5'AATCCAGAAACGATTGAAATGCTC and R 5' CTAAGTCATTTCATCACAATAATCT), obtained from the *R. breviuscula* and *R. pubera* genomes. In addition, a Tyba oligo-probe directly labeled with Cy3-dUTP was used (Marques *et al.*, 2015); Rb*-copia* (F 5'GGCAATTTGGAAGAGGATGT and R 5'GCTCCCACTGATCCTTTTGT) and Rb-*gypsy* (F 5'GGAGCATTAGAAAGCCCAAA and R 5'TGATTTTGTTCCTG GAGCAG) were also used. Probes were labeled by PCR using biotin-16-dUTP. The p*Ta*71 clone containing a 9kb *Eco*RI 45S fragment of *Triticum aestivum* ribosomal DNA (Gerlach and Bedbrook, 1979) was isolated by mini-prep and labeled with digoxigenin-11-dUTP by nick translation. For FISH, a mixture of 30 µl containing 100% formamide (15 µl), 50% polyethylene glycol (6 µl), 20× SSC (3 µl), 100ng of calf thymus DNA (1 µl), 10% SDS (1 µl), and 100ng of probe (4 µl) was treated at 70 °C for 10min, placed on ice, and immediately applied to the samples. Denaturation and hybridization were performed at 95, 50, and 38 °C, for 10min each, followed by 37 °C overnight in a humidified chamber. Post-hybridization washes were carried out in 6× SSC and 4× SSC/0.2% Tween-20 at room temperature. Probes labeled with biotin were detected with avidin–FITC, while those labeled with digoxigenin were detected with anti-dig–rhodamine diluted 1:100 in 2% BSA plus 4× SSC/0.2% Tween-20 (w/v). Post-detection washes were carried out in 4× SSC/0.2% Tween-20 at room temperature. Slides were mounted in 23 µl of the DABCO solution described above with 2 µl of 2 µg ml^–1^ DAPI.

### Detecting nuclear degeneration and PCD

Immunolabeling of pseudomonads with anti-5-methylcytosine (5-mC) was performed as described in Marques *et al.* (2011). First, anthers were fixed in absolute ethanol:glacial acetic acid (3:1, v/v). Preparations were squashed in 60% acetic acid followed by denaturation in a solution containing 50% formamide in 2× SSC. Afterwards, the preparations were washed in 1× PBS followed by blocking in 3% BSA in 1× PBS. Samples were incubated with 25ml of a solution (1:100, v/v) of mouse anti-5-mC (Eurogentec, cat. no. MMS-900P-A) antibody in 1% BSA in 1× PBS overnight at 4 °C followed by detection of signals with FITC–sheep anti-mouse antibody (Vector).

The integrity of degenerative nuclei was evaluated with the comet assay. Pseudomonads were isolated from 10 anthers of different sizes and placed in a 1.5ml microfuge tube containing a modified LB01 buffer (Doležel *et al.*, 1989) composed of 15mM Tris, 5mM EDTA, 80mM KCl, 20mM NaCl, 0.01% β-mercaptoethanol, 20mM MgCl_2_, and 0.05% polyvinylpyrrolidone (PVP) (w/v). Nuclei were removed by breaking the cell walls using a slight vortex agitation, and filtered through a 50 µm nylon layer. A 20 μl aliquot of the solution of nuclei was added to 120 μl of 1.5% low melting point (LMP) agarose in 1× PBS, pH 7.4 and, after a brief homogenization, samples were applied onto a slide covered with 1.5% agarose, in the same buffer. Slides were submitted to electrophoresis at 12V/130 mA for 1h. Samples were washed twice in 1× PBS, dehydrated in 100% ethanol, and then air-dried. Nuclei were stained with 50 μl of 2.5 μg ml^–1^ propidium iodide and observed using an epifluorescence microscope.

Flow cytometry was used to detect variation in the DNA amount in the degenerative nuclei. As control, nuclei of young leaf tissue of *R. pubera* (2C=3.53 pg, Marques *et al.*, 2015) was used as reference, and tested together with leaf nuclei of *R. breviuscula.* Nuclei were isolated in a Petri dish above ice in the modified LB01 buffer described above. Nuclei were filtered using a 50 µm nylon mesh, treated with 50 µg ml^–1^ RNase, and stained with 50 µg ml^–1^ propidium iodide. The measurements were conducted using the BD Accuri C6 flow cytometer, following the specifications of the manufacturer. For investigating the reduction of DNA content during the cell degeneration process, anthers of *R. breviuscula* were carefully dissected in modified LB01 buffer to obtain isolated pseudomonads. After collecting pseudomonads, nuclei were released using a mild vortexing, filtered through a 50 µm nylon layer, and stained with 50 µg ml^–1^ propidium iodide moments before measurement in a flow cytometer. Fifty thousand nuclei were measured in each of the five rounds.

For TEM, anthers were processed using modified Karnovsky’s solution (Karnovsky, 1965) and embedded in Araldite resin^®^ as described above. Ultra-thin sections (~70nm) of 10 anthers were collected in a copper grid and stained with 9% uranyl acetate and Reynold’s lead citrate solution (Reynolds, 1963). Sections were analyzed using a FEI Tecnai 12 transmission electron microscope at 60kV. Images were acquired with Analysis FEI software.

## Results

### Pseudomonad development

**Fig. 1.**
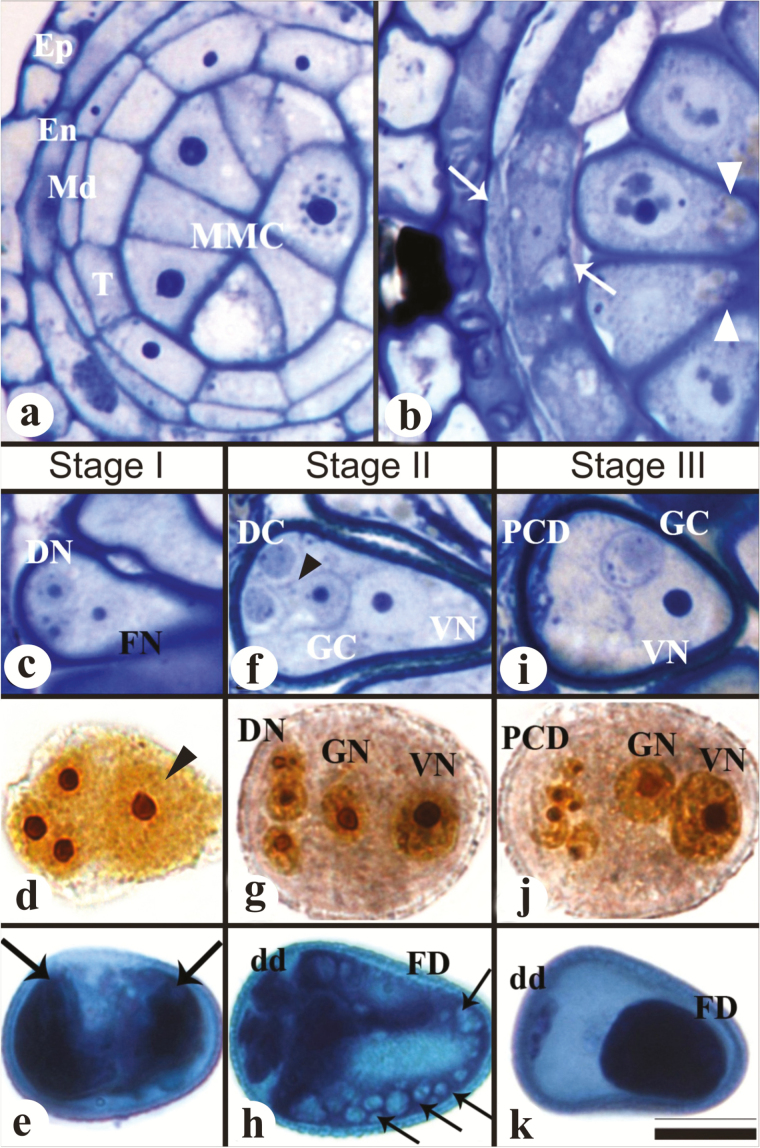
Pseudomonad development in *R. breviuscula*. (a–c, f, i) Semi-thin transversal sections of anthers stained with toluidine blue. (d, g, j) Squashed pseudomonads stained with Ag(NO)_3_. (e, h, k) Squashed pseudomonads stained with Alexander stain. (a) Young anther showing all five tissues (epidermis, Ep, endothecium, En, middle layer, Md, tapetum, T; and microspore mother cells, MMCs) still intact. (b) During entry of the MMC into prophase I, the anther exhibits PCD features in the middle layer and tapetum, which is represented by a dense shrunken cytoplasm packed with small vacuoles (arrows), when compared with the previous stage in (a). Vacuoles are also seen in the pseudomonad adaxial region (arrowheads). (c) Stage I of pseudomonad development, representing the end of meiosis. Two degenerative nuclei (DN) in the abaxial region and one functional nucleus (FN) in the center can be seen. (d) Note the group of four nuclei with nucleoli located at the central–abaxial region of the MMC. The arrowhead points to one slightly displaced nucleus. (e) Observe that, by this stage, two similar functional domains are present (arrows). (f) Stage II representing the end of first pollen mitosis (PM I). Note the presence of the generative cell (GC) in the center of the pseudomonad. The weakly stained vegetative nucleus can be seen in the adaxial region (VN). Degenerative cells are also present (DC). The arrowhead indicates the presence of a phragmoplast. (g) In this stage, the three smaller degenerative nuclei (DN) still present active nucleoli, similar to the vegetative nucleus (VN) and generative nucleus (GN). (h) Two cytoplasmic domains can be observed: (1) the reduced abaxial domain (degenerative domain; dd) and (2) the large adaxial domain (functional domain; FD) with several vacuoles (arrows). (i) Stage III of development representing the immature pollen grain containing debris of the degenerative cells (PCD). The generative cell (GC) is located beside the weakly stained vegetative nucleus (VN). Degenerative nuclei exhibit features of PCD degeneration (j) and only debris of the degenerative domain (dd) can be observed in the abaxial region (k). Scale bar in (k), also for (a–j)=10 µm. (This figure is available in colour at *JXB* online.)

To check the overall structure of pseudomonads, young anthers of *Rhynchospora* were analyzed by light microscopy. Anthers are composed of five tissues: epidermis, endothecium, middle layer, tapetum, and sporogenic tissue with microspore mother cells (MMCs) (Fig. 1a). During prophase I, tapetum cells exhibited differential cytoplasm staining in relation to early pre-meiotic stages, and MMCs presented a nucleus facing the abaxial region, next to the tapetum, with a visible accumulation of vacuoles in the adaxial region of the cell (Fig. 1b). Pseudomonads were characterized in three stages of development (Fig. 1c–k).

In stage I, corresponding to the end of meiosis II, pseudomonads exhibit degenerative cells displaced to the abaxial region while the functional cell appears centrally positioned (Fig. 1c, d). Cytoplasm and cell walls were weakly stained by silver nitrate, and cells presented evident nuclei and nucleoli (Fig. 1d). Alexander staining revealed two equal cytoplasmic domains (Fig. 1e). Stage II is characterized by the occurrence of PM I, when the functional nucleus gives rise to generative and vegetative nuclei. In this stage, degenerative cells were still present, clearly delimited by the phragmoplast (Fig. 1f), bearing smaller nuclei in comparison with stage I (Fig. 1g). Generative and vegetative nuclei also displayed nucleoli that appeared to be active (Fig. 1g) at this stage, as did degenerative nuclei. Alexander staining revealed two unequal cytoplasmic domains: a smaller degenerative domain in the abaxial region and a larger functional domain filled with vacuoles in the adaxial region (Fig. 1h). In the overview of stage II pseudomonads using TEM, it was possible to observe the clear difference in the cytoplasm sizes between degenerative and functional domains (Supplementary Fig. S1a, b at *JXB* online). Stage III represents the immature pollen grains, which exhibited degenerative cells with PCD features such as cytoplasm contractions, cellular debris in the abaxial region (Fig. 1i, k), and nuclear fragmentation (Fig. 1j). Vacuoles accumulated and occupied the region between the degenerative domain and the generative cell, allocating the generative cell to the adaxial position (Supplementary Fig. S2a, b). Despite this repositioning and its small size, the generative cell retained a cytoplasm rich in organelles (Supplementary Fig. S2c), unlike degenerative cells. The generative nucleus was seen next to the vegetative nucleus (Fig. 1i, j), both with nucleoli, and with a large functional cytoplasmic domain in the adaxial region (Fig. 1k). All of the cells were clearly delimited by a cell wall (Supplementary Fig. S2d, e).

### Establishment of cell asymmetry and the role of cytoskeleton elements

**Fig. 2.**
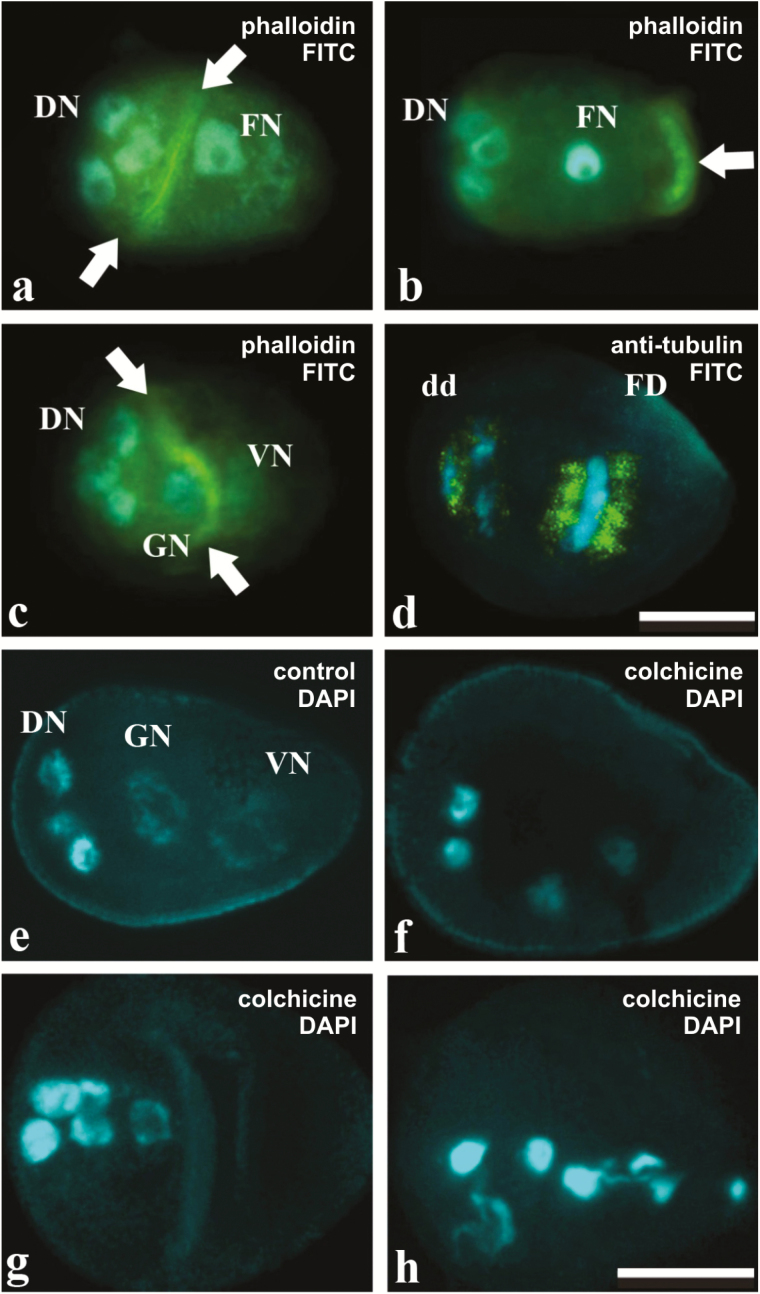
Behavior of the cytoskeleton in pseudomonads of *Rhynchospora*. (a–c) F-actin detection using phalloidin-FITC (stronger signal) and nuclei counterstained with DAPI . Degenerative nuclei (DN) are positioned in the abaxial region. (a) Stage I pseudomonads in telophase II with more intense fluorescence signals (arrows) between the degenerative and functional nuclei (FN). (b) Pseudomonad, still in stage I, but after meiosis, showing no intense phalloidin-FITC signals between degenerative nuclei and the functional nucleus (FN), with slightly more intense signals in the adaxial region (arrow), relative to vacuole accumulation (see arrowhead in Fig. 1b). (c) Stage II pseudomonads treated with phalloidin-FITC. Note an abundance of FITC bright signals (arrows) between generative (GN) and vegetative nuclei (VN). (d) Immunodetection of tubulin and nuclei counterstained with DAPI. Stage II pseudomonad treated with anti-tubulin (stronger signal). Note a separation of generative and vegetative nuclei, and few signals in the degenerative domain. Scale bar=10 µm. (e–h) Depolymerization of microtubules using colchicine treatment. Nuclei are stained with DAPI. (e) Stage II pseudomonads with commonly observed features, unaffected by colchicine. (f–h) Pseudomonads affected by colchicine treatment. (f, g) Coenocytic cells with four and five morphologically similar nuclei in the central–abaxial region, respectively. Observe that these nuclei resemble the degenerative nuclei. (h) In a more advanced stage, the coenocytic cell shows five nuclei with fragmentation features. Scale bar in (d) =10 µm, such as in (h), (a–c), (e–g)=10 µm. (This figure is available in colour at *JXB* online.)

To determine whether the actin cytoskeleton is involved in the expression of cell asymmetry, we used phalloidin–FITC to mark F-actin. In stage I, phalloidin–FITC signals appeared either as an agglomerate associated with the delimitation of degenerative and functional domains during telophase II (Fig. 2a), or as a discrete accumulation in the adaxial region, corresponding to vesicle accumulation in this area after meiosis (Fig. 2b). In stage II, after PM I, phalloidin–FITC was detected again as an agglomerate correlating with the functional separation between generative and vegetative nuclei (Fig. 2c). In order to check for differential spindle assembly, we applied anti-tubulin antibody to the pseudomonads. The spindle assembly was observed in the functional cell during separation of generative and vegetative nuclei, while irregular spindle formation was observed surrounding degenerative cells (Fig. 2d).

Analysis of 358 colchicine-treated cells indicated that 291 cells (82%) exhibited the commonly observed features of pseudomonads (Fig. 2e), while 67 cells (18%) exhibited morphological features related to pseudomonad disruption, probably due to microtubule disorganization. These cells presented four (Fig. 2f) to five (Fig. 2g) morphologically similar nuclei, close to one another, in the abaxial to central region of what appears to be a coenocytic cell, with no evidence of cell plate organization. All nuclei appeared more condensed, similar to the degenerative nuclei of control pseudomonads, and, sometimes, highly disorganized, exhibiting features of degeneration (Fig. 2h).

### Repetitive DNA families and pseudomonad development

In order to identify high-copy-number sequences for nuclear characterization in developing pseudomonads, we sequenced and annotated 45 fosmids, each containing an average of ~40 kbp of *R. breviuscula* genomic DNA. We could assemble 921 contigs larger than 200bp in length that contained long terminal repeat (LTR) retrotransposons (54.4%), transposons (27.0%), non-LTR retrotransposons (10.1%), low complexity DNA (1.0%), rDNA (2.7%), and other sequences (4.8%). *Ty*1-*copia*-like and *Ty*3-*gypsy*-like elements represented a respective ~58% and ~36% of all LTR retrotransposons, while ~6% could not be assigned to a superfamily and remained unclassified. We also found three scaffolds containing AT-rich repeats with a length of 172bp. The scaffold named Rbsat33246 (33 246bp length) showed 189 copies of this satellite repeat, which exhibits high similarity to the holocentromeric Tyba repeat of *R. pubera* (Marques *et al.*, 2015). Fragments of 45S rDNA from *T. aestivum* (p*Ta*71 clone), as well as identified reverse transcriptases of *copia* (Rb-*copia*, 246bp), *gypsy* (Rb-*gypsy*, 561bp), and Tyba-like sequences (172bp), were selected for FISH. Alignments can be seen in Supplementary Fig. S3.

**Fig. 3.**
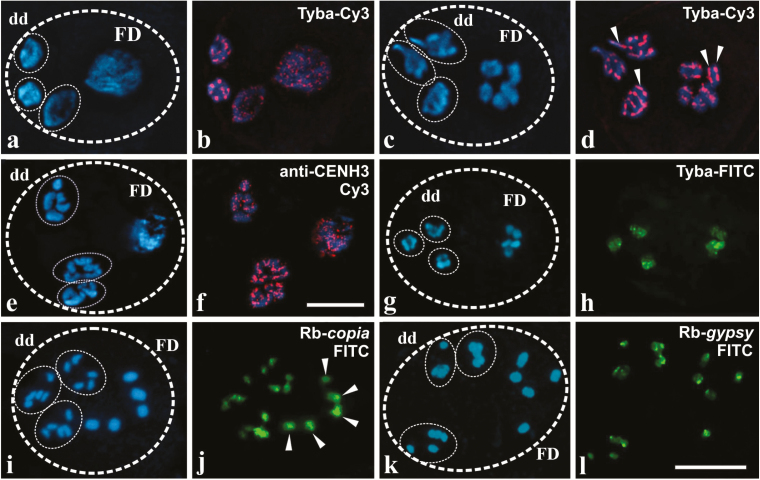
Localization of repetitive elements by fluorescent *in situ* hybridization (a–d, g–l) and CENH3 by immunostaining (e–f) in *Rhynchospora* pseudomonads. (a–d) FISH using the Tyba repeat on pseudomonads of *R. pubera* (stronger signal). Nuclei and chromosomes counterstained with DAPI. (a, b) In stage I, fluorescent signals can be seen scattered around degenerative nuclei and the functional nucleus. Note that degenerative nuclei tend to present more localized signals. (c, d) During PM I in stage II, hybridization signals are seen in a linear manner, occurring through the entire extension of the chromosomes. Note that the functional domain chromosomes present two sets of hybridization signals, indicating replication, while the degenerative domain chromosomes present only one set of hybridization signals, meaning that these chromosomes were not replicated (arrowheads). (e, f) Immunostaining of CENH3 on stage II pseudomonads of *R. pubera* (stronger signal) suggests that these centromeric proteins are co-localized with Tyba. Scale bar in (f), also for (a–e)=10 µm. (g–l) FISH using three different repetitive DNA probes on stage II pseudomonads of *R. breviuscula* (stronger signal). FISH using Tyba-like probes in *R. breviuscula* showing that hybridization signals appear broadly dispersed through the chromosomes in PM I (g, h). FISH signals of the Rb-*copia* probe are smaller and less intense in the degenerative domain in relation to the chromosomes of the functional domain (arrowheads; see Supplementary Figure S4m for a less saturated capture) (i, j), while FISH signals for the Rb-*gypsy* probe remains similar in both domains (k, l). Scale bar in (l), also for (g–k)=10 µm. (This figure is available in colour at *JXB* online.)

Both *Rhynchospora* species exhibited 2*n*=10 holocentric chromosomes (see Fig. 3; Supplementary Fig. S4). The 45S rDNA probe showed two terminal FISH signals in mitotic chromosomes for *R. breviuscula* (Supplementary Fig. S4a) and only one equal-sized hybridization signal in each nucleus of both functional and degenerative cells of stage I pseudomonads (Supplementary Fig. S4b). In contrast, signals for the centromeric Tyba repeat were brighter in the condensed degenerative nuclei than that of the functional nucleus in *R. pubera* and *R. breviuscula* in pseudomonads of the same stage (Fig. 3a, b and Supplementary Fig. S4i–k, respectively). The Rbsat33246 probe (Tyba-like) did not show a perfect line-like structure in *R. breviuscula* chromosomes in interphase and metaphase of mitosis (Supplementary Fig. S4c–f), or in bivalents of meiosis (Supplementary Fig. S4g, h), as observed in *R. pubera* chromosomes (Figs 3c, d). Nonetheless, we did not find a significant loss in the hybridization signals of degenerative chromosomes in stage II pseudomonads in relation to the functional domain in both cases (Fig. 3a, b, g, h ; Supplementary Fig. S4l, m). Assaying the different stages of *R. pubera* pseudomonad development with the centromeric Tyba repeat showed that, despite differential cell domains, the holocentromere-typical distribution of centromeric repeats occurred in all four cells. The degenerative chromatin showed linear Tyba FISH signals along chromosomes, with similar brightness to the chromosomes of the functional domain (Fig. 3c, d). This observation reinforces the idea that differences seen in the hybridization signal between degenerative and functional nuclei could be due to the state of chromatin condensation in each domain. It also clearly shows that *R. pubera* chromosomes in the functional domain exhibit two line-like signals after FISH with the Tyba probe, while degenerative domain chromosomes exhibited only one line (see arrowheads in Fig. 3c and d). This observation indicates that chromosomes in the functional domain are replicated and chromosomes in the degenerative domain are not. Immunodetection using anti-CENH3 antibody appears to have confirmed the holocentric organization of chromosomes (Fig. 3e, f).

LTR retrotransposon probes were also used to study differences between degenerative and functional nuclei in stages I and II. A conserved region of the reverse transcriptase gene, exhibiting ~50% identity with members of several *copia* lineages (Oryco, Sire, Retrofit, and Tork) in various grass species, was used as a FISH probe. In somatic interphase nuclei, *copia* signals were more prominent in some chromocenters, and signals were more intense at the ends of somatic chromosomes (Supplementary Fig. S5a–f). When we used the same probe on stage I pseudomonads, degenerative nuclei showed stronger signals, although in both degenerative and functional domains signals were associated with more condensed chromatin (Supplementary Fig. S5g). On the other hand, chromosomes undergoing PM I in stage II pseudomonads revealed a visible difference in the signal intensity between degenerative and functional domains (Fig. 3i, j; and a less saturated image in Supplementary Fig. S5h, i). A segment of the *gypsy* superfamily reverse transcriptase gene was also used as an additional probe. This FISH probe exhibited ~80% identity with members of several chromovirus lineages (Del, Reina, CRM, and Galadriel) in grasses, but not with the Athila/Tat clade. In contrast to the unequal distribution of *copia* signals, *gypsy* signals were detected in the terminal chromosome regions without evident differences between degenerative and functional domains of stage II pseudomonads (Fig. 3k, l). In interphase nuclei of *R. breviuscula*, the *gypsy* probe showed signals adjacent to chromocenters (Supplementary Fig. S5j, k). The varying distribution of LTR retrotransposons could be caused by an early loss of *copia* repeats in degenerative chromosomes. However, it is important to note that, unlike *copia*, for which possibly all major LTR retrotransposon lineages were detected by FISH, the *gypsy* probe did not identify members of Athila/Tat clades.

### The degenerative domain is marked by intense cytosine methylation and DNA fragmentation

**Fig. 4.**
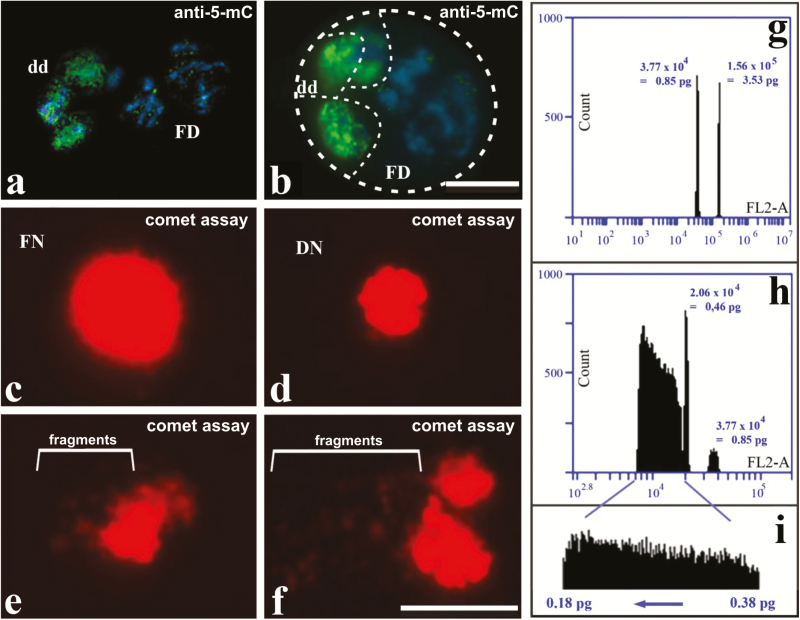
Characterization of degenerative nuclei in pseudomonads using immunostaining of 5-methylcytosine (5-mC) in *R. pubera* (a, b), and the comet assay (d–f) and flow cytometry (g–i) in *R. breviuscula*. (a) Stage II pseudomonads during PM I with degenerative chromosome sets showing stronger 5-mC DNA signals. (b) These signals are also present in degenerative nuclei after PM I. Scale bar in (b), also for (a)=10 µm. (c) Note that the functional nucleus (FN) does not exhibit evidence of DNA fragmentation after electrophoresis. (d–f) Degenerative nuclei (DN) indicating three stages of DNA fragmentation. Scale bar in (f), also for (c–e)=10 µm. (g) Flow cytometry of *R. breviuscula* and *R. pubera* leaf tissue. Two peaks can be observed: one with 1.56×10^5^ fluorescent units represents 2C nuclei of *R. pubera*, which have a known C-value of 3.53 pg. The second peak, with 3.77×10^4^ fluorescent units, represents *R. breviuscula* 2C nuclei. Based on the fluorescence value of *R. pubera* nuclei, it can be concluded that the DNA content of *R. breviuscula* is 2C=0.85 pg. (h) Flow cytometry of *R. breviuscula* pseudomonad haploid nuclei. Note that a small 2C peak can be observed again and, next to it, another peak is seen with 2.06×10^4^ fluorescent units, representing haploid nuclei with C=0.46 pg. A large heterogeneous smear is also observed, representing a diverse population of nuclei with C-values <0.46 pg. (i) The heterogeneous DNA values, extracted from (h) above, cover a range from 0.18 pg to 0.38 pg, indicative of DNA degradation from the 0.46 pg haploid state. (This figure is available in colour at *JXB* online.)

The distribution of 5-mC was tested to determine whether the observed differences in degenerative and functional chromatin organization are accompanied by changes in the DNA methylation status. At stage II, during and after PM I, both nuclei and chromosomes of the degenerative domains were hypermethylated, while the chromatin of the functional domain appeared slightly methylated (Fig. 4a, b). To check for signals of DNA fragmentation, we performed a comet assay using nuclei isolated from *R. breviuscula* pseudomonads. After electrophoresis, undersized (significantly less than 10 µm diameter) nuclei were observed, and these exhibited small migrating fragments (Fig. 4d–f), while this was not observed in the large functional nuclei (Fig. 4c). Flow cytometry was used to determine whether nuclei of pseudomonads experience loss of DNA. Initially, a control experiment was done using nuclei obtained from leaves to confirm the C-value of *R. breviuscula*. Two peaks were clearly identified, one corresponding to *R. pubera* diploid nuclei (2C=3.53 pg) and the other to *R. breviuscula* diploid nuclei at 0.85 pg (Fig. 4g; Supplementary Table S1). To check DNA C-value differences between degenerative and functional nuclei, pseudomonads of *R. breviuscula* in distinct developmental stages were isolated and used in flow cytometry. Three peaks could be observed: (i) 0.85 pg relative to replicated chromatin of a functional nucleus before PM I; (ii) 0.46 pg for haploid nuclei, relative to unreplicated chromatin of all cell domains; and (iii) a range of values from 0.38 pg to 0.18 pg, relative to fragmented chromatin of degenerative cells (Fig. 4h,i; Supplementary Table S1).

**Fig. 5.**
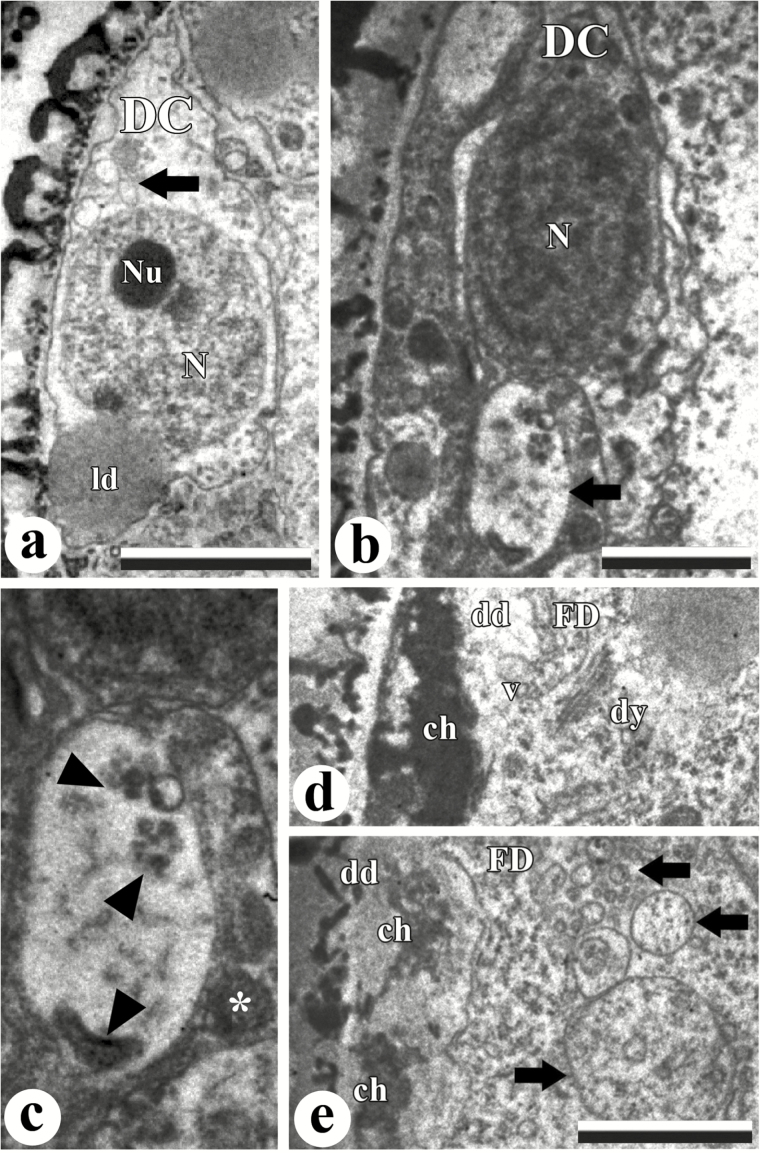
Ultrastructural features of cell death and DNA fragmentation in *R. breviuscula* pseudomonads. (a) Degenerative cell (DC) with no evident signs of PCD. Observe the electron-light cytoplasm containing an organelle-rich region with small vacuoles (arrow) and a large lipid droplet (ld). Magnification ×5800. Scale bar=2 µm. (b) Degenerative cell (DC) in early PCD. Note the more electron-dense cytoplasm and the nucleus with condensed chromatin (N). Magnification ×5800. Scale bar=1 µm. This cell also exhibits an evident lytic vacuole (arrow) containing electron-dense materials. The higher magnification image (magnification ×13 500) in (c) shows more details of a lytic vacuole (arrowheads), suggesting a degradation process, and the cytoplasm with other organelles next to it (*). (d, e) The interface between degenerative (dd) and functional domains (FD) in the stage III pseudomonads in advanced PCD, showing very condensed chromatin (ch) with signs of fragmentation in the degenerative cells. Dictyosomes (dy) associated with membranes and small vacuoles (v) at the edge of the functional domain are present (d). Arrows indicate large vacuoles in the functional domain (e). Organelles are rarely observed in dd in this stage. Magnification ×13 500 in (d) and (e). Scale bar in (e), also for (d)=1 µm.

The organization of stage II and III pseudomonads was further analyzed by TEM. During stage II, before the PCD process, degenerative cells exhibited functional cytoplasm containing organelles and a nucleus with diffuse chromatin and an intact nucleolus (Fig. 5a; Supplementary Fig. S1a, b). Small vacuoles were identified in this stage (Fig. 5a). In stage III, at early stages of PCD, these cells showed an electron-dense cytoplasm, more condensed chromatin, and a large vacuole containing cytoplasmic inclusions that made it morphologically similar to lytic vacuoles (Fig. 5b, c). In stage III, a more advanced stage of PCD, the degenerative domains exhibited morphological differences in relation to the functional domain. At first, the chromatin was strongly condensed (Fig. 5d) or disorganized/fragmented and almost entirely lost (Fig. 5e). Organelles were absent from degenerative domains, while the functional cell cytoplasm showed evidences of autophagic activity, including dictyosomes and vacuoles adjacent to degenerative domains (Fig. 5d, e).

## Discussion

In early stages of anther development of *Rhynchospora*, the morphology of these was similar to that found in other higher plants, with the five typical tissues described for monocots (Bedinger, 1992). However, the sporogenous tissue exhibits a different organizational profile, with the development of an asymmetrical tetrad (pseudomonad), that is described for Cyperaceae (Maheshweri, 1950; Ranganath and Nagashreee, 2000). In *R. breviuscula* and *R. pubera*, three degenerative nuclei occupy the abaxial region and a functional nucleus appears located in the center of the pseudomonad. This arrangement is similar to the one reported in other *Rhynchospora* species (Tanaka, 1941; Coan *et al.*, 2010; San Martin *et al.*, 2013), but different from that reported for other genera of Cyperaceae. In species of *Bulbostylis*, *Carex*, *Cyperus*, *Eleocharis*, *Fimbristylis*, *Kyllingia*, *Scirpus*, and *Scleria*, the degenerative nuclei occupy the adaxial region of the pseudomonad (Tanaka, 1941; Padhye, 1968; Kirpes *et al.*, 1996; Furness and Rudall, 1999; Brown and Lemmon, 2000).

The polarized pseudomonad of *R. breviuscula* showed degenerative domains that appear to be viable and physiologically active when tested by silver nitrate, Alexander staining, and TEM. However, the results also indicated that these domains fail to complete cell division in stage II of PM I, as was previously reported in *R. pubera* (San Martin *et al.*, 2013). TEM indicated that asymmetric cell division results in an unequal distribution of cytoplasm between functional and degenerative cells. In *Carex blanda*, evidence of polarity establishment is observed in early prophase I. In *Rhynchospora*, the vacuoles accumulated at the cellular adaxial pole could be evidence of this polarity. During anaphase II/telophase II, cytoskeleton elements appear to be responsible for the displacement between functional and degenerative sets (Brown and Lemmon, 2000). Assays performed with phalloidin–FITC in *R. breviuscula* and immunodetection of tubulin in *R. pubera* confirmed the importance of the cytoskeleton in domain delimitation and establishment of cell asymmetry throughout pseudomonad development. This was also reported in *R. pubera* using other immunocytochemical procedures (San Martin *et al.*, 2013).

Although degenerative cells in *Rhynchospora* may start a cell division, there was no evidence that they completed cytokinesis, in disagreement with observations or predictions in the literature for other Cyperaceae (Brown and Lemon, 2000; Ranganath and Nagashree, 2000). Tubulin immunostaining showed that a normal spindle assembly occurs only in the functional cell of *R. pubera*. In contrast, *C. blanda* forms a regular spindle assembly in all cells (Brown and Lemon, 2000). These differences demonstrate that processes leading to pseudomonad formation in this family are evolutionarily dynamic.

While it is clear that the cytoskeleton plays a strong role in the establishment of different cell lines in pseudomonads, it was not known what consequences might result from disruption of the pre-prophase band and/or the phragmoplast. The treatment of *R. breviuscula* anthers with colchicine inhibited the usual cell delimitation, and revealed pseudomonads with four undifferentiated nuclei, comparable with degenerative nuclei found in the control. This test showed that delimitation of different cytoplasms must be a decisive event, enabling regular gametogenesis of one meiotic product in the Cyperaceae context. The same colchicine treatment also resulted in the presence of five nuclei in some pseudomonads, which could be explained by karyokinesis of one nucleus after meiosis. This is likely to be a product of the functional nucleus, since (i) it is uncommon for degenerative nuclei to undergo karyokinesis (Ranganath and Nagashree, 2000); (ii) FISH using a centromeric Tyba repeat confirmed that, in *R. pubera*, degenerative nuclei do not undergo replication; and (iii) degenerative nuclei never generated daughter nuclei in control pseudomonads of *R. breviuscula*. These results also indicate that the replication of a single meiotic product defines the functional nucleus under normal conditions (without colchicine treatment), because even in a homogeneous environment (with colchicine treatment), only one nucleus is fully able to complete karyokinesis. The functional nucleus and degenerative nuclei could already be defined at an earlier stage, and this may not be a stochastic event. According to Cabral *et al.* (2014), 19.5% of all meiosis II products of *R. pubera* had irregular chromosome numbers. This could be an explanation for the preferential selection of functional cells. In this context, it is possible that, during anaphase II, chromosome sets without rearrangements could be selected to retain replication capability and compose the functional cell via meiotic drive, as suggested by Furness and Rundall (2011).

A common feature of many Cyperaceae species, such as *Rhynchospora*, *Carex*, and *Eleocharis*, is the shortening of chromosomes in the degenerative domain during pseudomonad development (Harms, 1972; Hoshino and Shimizu, 1986; Luceño *et al.*, 1998; Da Silva *et al.*, 2005; San Martin *et al.*, 2013). FISH with repetitive DNA probes was used to test if the differences in chromosome size could be due to loss of repetitive sequences. FISH in *R. pubera* and *R. breviuscula* using the Tyba repeat and in *R. breviuscula* using a *gypsy* probe showed no major difference in signal intensity, indicating that these sequences are not primarily responsible for discrepancies in sizes between chromosomes in the degenerative and functional domains. Tyba interacts with CENH3 along the chromosomes of *Rhynchospora* (Marques *et al.*, 2015), and the same seems to occur in the chromosomes of degenerative cells. Another interesting fact is that Tyba repeats were not replicated in degenerative nuclei, suggesting that the nuclear DNA is unreplicated when degenerative cells initiate mitosis. This fact, together with the assembly of abnormal spindles, might be the trigger to initiate PCD, resulting in an event known as mitotic disorder (Castedo, 2004).

FISH with *copia* retrotransposon probes showed that signals were reduced in the degenerative sets compared with the functional set, in stark contrast to the retained *gypsy* repeats. San Martin *et al.* (2013) showed an early elimination of telomeric motifs in stage I pseudomonads of *R. pubera*, and the same may possibly be occurring for *copia* retrotransposons. The preferential loss of *copia*-like retrotransposons suggests an independent fate for each retrotransposon superfamily, which may be related to their very different insertion and accumulation specificities (Bennetzen and Wang, 2014). We assume that the degenerative chromosome shortening could be due to (i) a lack of DNA replication prior to PM I initiation, as proposed in *Carex ciliato-marginata* (Hoshino and Shimizu, 1986) and observed in *R. pubera* for Tyba; and/or (ii) a non-stochastic elimination process, as seen for *copia* in *R. breviuscula* (our study) and telomeric motifs in *R. pubera* (San Martin *et al.*, 2013). Although deletions of transposable elements can be responsible for alterations in the nuclear DNA content in short time periods (Ma *et al.*, 2004; Vitte and Panaud, 2005), further investigations are needed to clarify the mechanisms involved in the reduction of repetitive motifs in degenerative chromosomes. The 45S rDNA is not among the repetitive elements that are lost early in the process. Silver nitrate staining indicated that degenerative nuclei have apparently functional nucleoli until late stages of pseudomonad development, a phenomenon that may be necessary to support PCD metabolism.

PCD processes in degenerative cells seem to initiate shortly after the chromosome diminishment observed in PM I, when chromatin exhibited signals of DNA hypermethylation and condensation. These features were also associated with dense cytoplasm and large lytic vacuoles. These vacuoles were morphologically similar to those found in suspensor cells and tracheal elements in the process called ‘vacuolar cell death’ (VCD) (van Doon *et al.*, 2011). Three features described by van Doorn *et al.* (2011) as typical of VCD were observed in pseudomonads degenerative cells of *Rhynchospora*. These were (i) a gradual decrease in cytoplasmic volume; (ii) an increase in the size and number of lytic vacuoles; and (iii) cargo degradation in the vacuolar lumen. Additionally, chromatin condensation with subsequent DNA fragmentation is also commonly associated with PCD (Staley *et al.*, 1997; Reape and McCabe, 2008). The elimination of the degenerative domain, associated with an increased richness of other vacuoles in the adjacent functional cytoplasm, suggests that the fate of degraded biomolecules is to be assimilated by the forming pollen grain in a co-ordinated and non-stochastic process. Although VCD seems to be the predominant kind of PCD occurring in degenerative cells, typical features such as the rupture of the vacuoles were not clearly observed. Choosing between the main PCD types described in plants, necrotic cell death or VCD (van Doon *et al.*, 2011), we believe that the cell death in pseudomonads of *Rhynchospora* is associated with vacuolar action. Both the comet assay and measurement of the amount of DNA using nuclei of pseudomonads showed a progressive DNA fragmentation before pollen grain formation in *R. breviuscula*. TEM, the comet assay which shows DNA fragmentation (Rigonato *et al.*, 2005), and nuclear flow cytometry for *R. breviuscula* all indicated that degenerative cells undergo a continuous process of cytoplasmic contraction, lytic vacuole action with reduction in organelle number, and chromatin fragmentation with a decrease in genomic DNA content.

In conclusion, the formation of only one pollen grain starting from one MMC is a multistep process. This event involves an unknown cellular signaling chain that (i) drives a cell selection enabling one nucleus to undergo replication and form the functional domain, and/or disabling replication in three nuclei making them degenerative; (ii) controls the movement of cytoskeleton elements, provoking an unequal displacement of nuclei to arrange an asymmetrical tetrad; (iii) unequally divides the cytoplasm of the cellular structure forming three smaller degenerative cells with nuclei lacking replication capability; (iv) attempts to perform cell division in degenerative cells with an unreplicated genome, which is followed by DNA methylation and chromatin fragmentation, including non-random elimination of repetitive DNA families; and (v) results in a particular case of PCD, presenting an intense vacuolar activity in the degenerative domain and in the functional domain adjacent to it. The exact mechanisms of DNA elimination and cytoplasmic dynamics during PCD are still unresolved, but these findings suggest new approaches for studying the physiology and chromatin fates of pseudomonads.

## Supplementary data

Supplementary data are available at *JXB* online.

Figure S1. Overview of *Rhynchospora* stage II pseudomonad ultrastructure.

Figure S2. Semi-thin TEM sections showing more details of differential organelle accumulation and membranes separating cytoplasmic environments.

Figure S3. Sequences of repetitive DNA families used as probes for fluorescent *in situ* hybridization (FISH).

Figure S4. FISH in *R. breviuscula* stage II pseudomonads using 45S rDNA and Rbsat33246 probes.

Figure S5. FISH in *R. breviuscula* using Rb-*copia* and Rb-*gypsy* probes.

Table S1. Results of flow cytometry experiments using pseudomonads of both *R. breviuscula* and *R. pubera.*

Supplementary Data
