# Supplementary material for: Developmental programmed cell death during asymmetric microsporogenesis in holocentric species of Rhynchospora (Cyperaceae)
Source: J Exp Bot. 2016 Aug 4;67(18):5391–401. doi: 10.1093/jxb/erw300 (PMC5049389; doi:10.1093/jxb/erw300)
Supplement: Supplementary Data [file supp_67_18_5391__index.html]

Developmental programmed cell death during asymmetric microsporogenesis in holocentric species of Rhynchospora (Cyperaceae) — Developmental programmed cell death during asymmetric microsporogenesis in holocentric species of Rhynchospora (Cyperaceae) — Supplementary Data 

# Developmental programmed cell death during asymmetric microsporogenesis in holocentric species of *Rhynchospora* (Cyperaceae)

## Supplementary Data

Data files

- supplementary\_figures\_S1\_S5\_table\_S1.pdf - Supplementary Data
