# Supplementary material for: Developmental programmed cell death during asymmetric microsporogenesis in holocentric species of Rhynchospora (Cyperaceae)
Source: J Exp Bot. 2016 Aug 4;67(18):5391–401. doi: 10.1093/jxb/erw300 (PMC5049389; doi:10.1093/jxb/erw300)
Supplement: Supplementary Data [file supp_erw300_supplementary_figures_S1_S5_table_S1.pdf]

Supplementary material

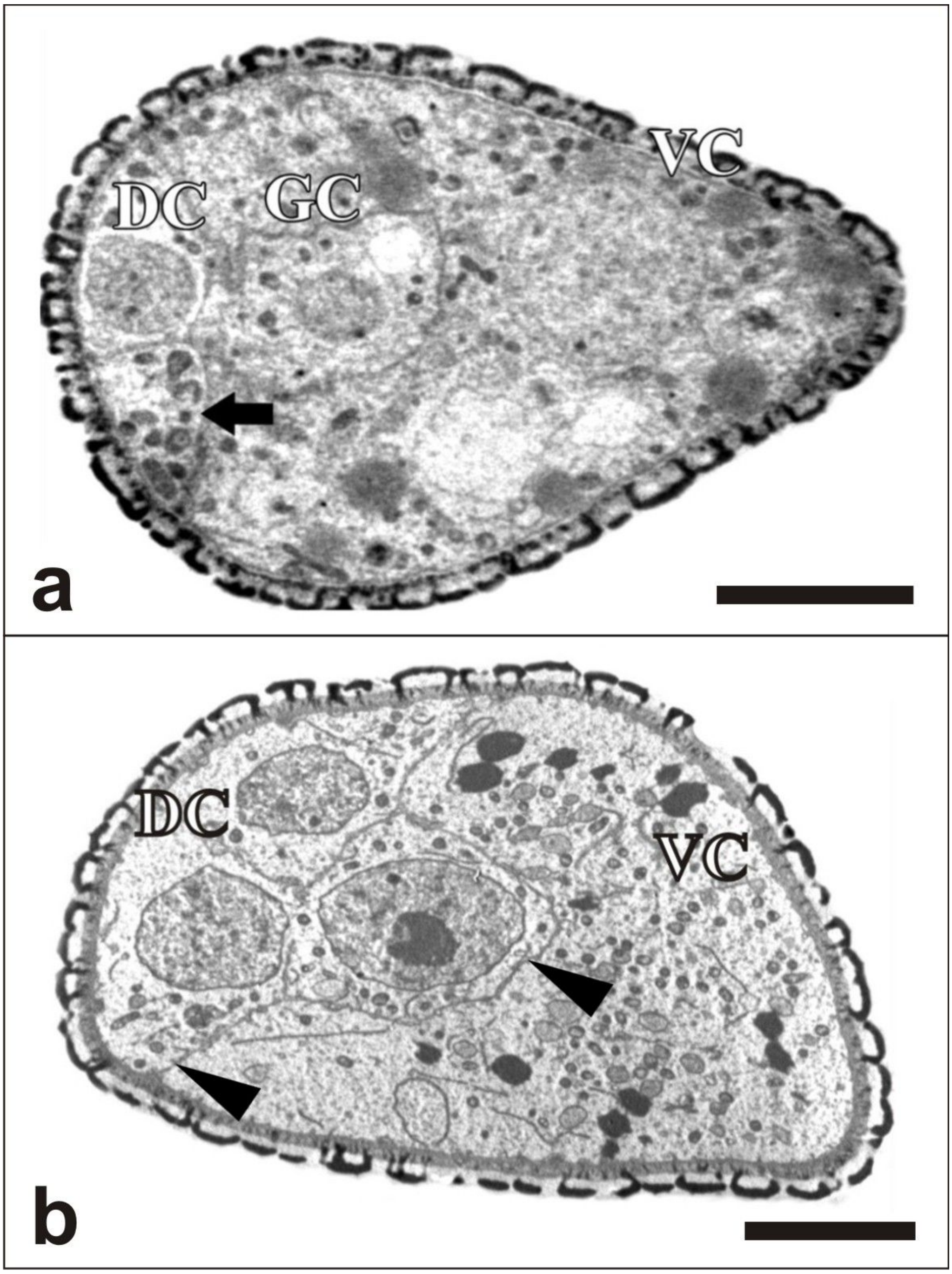

Figure S1. Ultrastructural features of *Rhynchospora* stage II pseudomonads, after PM I, in (**a**) *R. breviuscula* and (**b**) *R. pubera*. The functional domain of pseudomonads is composed of a generative cell (GC) and a vegetative cell (VC), and contains most of the cytoplasm volume. Degenerative cells (DC) exhibit small cytoplasmic spaces, but still contain organelles (arrow). Cells are delimited by the cell plate (arrowheads), which can be better observed in the Figs. S2d-e. In this stage, degenerative cell nuclei present diffuse and intact chromatin. Magnification 1,450×. Scale bars = 5 µm.

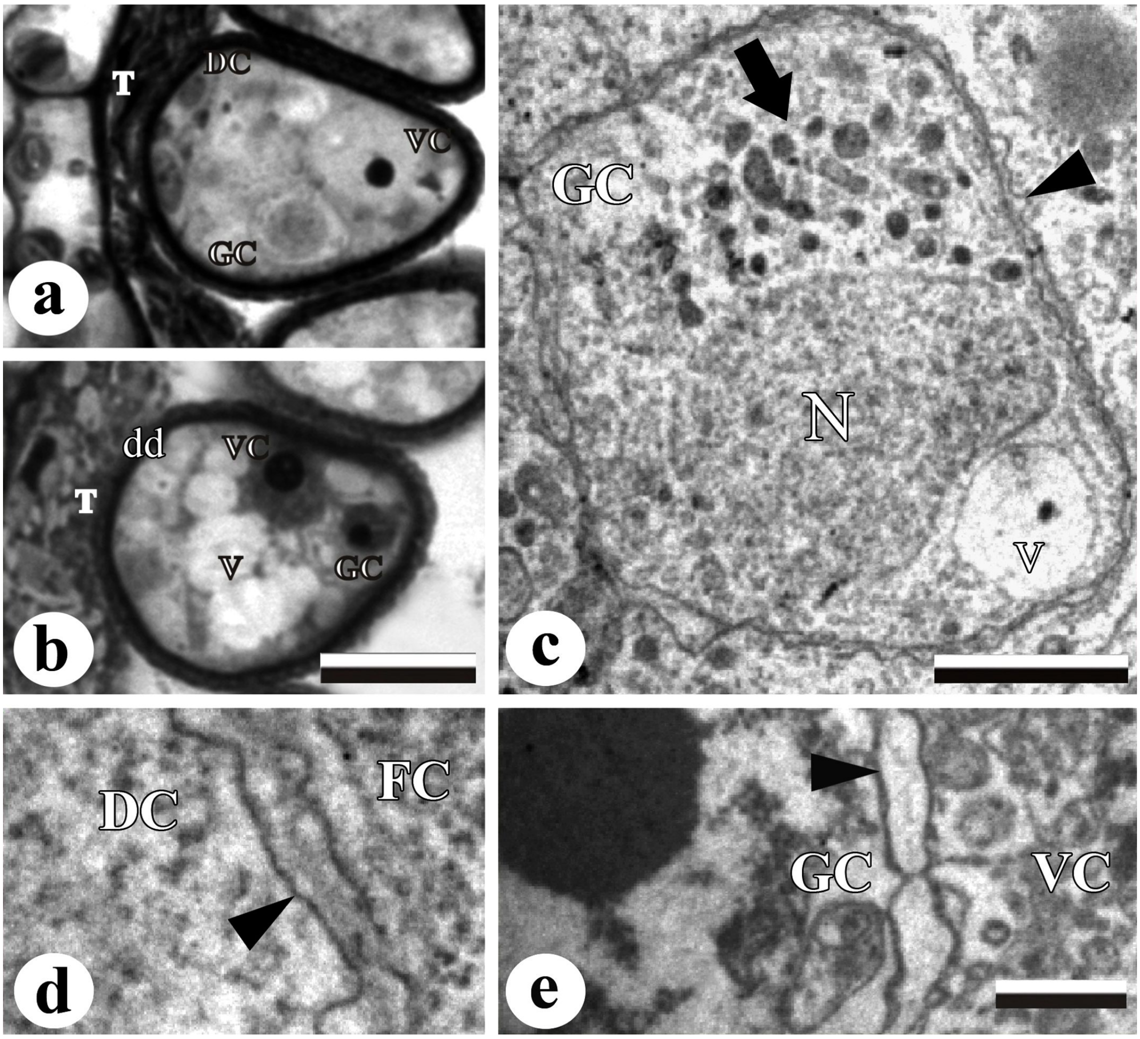

Figure S2. Structure and ultrastructure of *R. breviuscula* stage III pseudomonads. (**a-b**) Semi-thin transversal sections of anthers stained with toluidine blue, showing accumulation of some vacuoles (V) between degenerative cells or degenerative domains (DC or dd) at abaxial region next to the tapetum (T), and vegetative (VC) and generative (GC) cells. Scale bar in a and b = 10 µm. (**c**) Ultrastructural features of *R. breviuscula* generative cell (GC). This cell presents electron light cytoplasm filled with organelles (arrow), delimited by a cell plate (arrowhead). Observe a small vacuole (V) next to a nucleus with diffuse chromatin (N). Magnification 3,900×. Scale bar = 2 µm. (**d-e**) Closer view of cell plates (arrowheads) between degenerative cell (DC) and functional cell (FC) in **d,** and between generative cell (GC) and vegetative cell (VC) in **e**. Scale bar in e and d = 0.5 µm.

```
(A) Tyba-satDNA-like x RbSat33246
Score             Expect      Identities        Gaps        Strand
282 bits(312)     2e-81       156/156(100%)     0/156(0%)   Plus/Plus
RbSat33246      1-ATTGAAATGCTCAAATGATACTCAGAATTGCATTATTTGCACTTTATATGCGTAATTACTATGTATAATCCAATCTTTTT
                  ||||||||||||||||||||||||||||||||||||||||||||||||||||||||||||||||||||||||||||||||
Tyba-satDNA    17-ATTGAAATGCTCAAATGATACTCAGAATTGCATTATTTGCACTTTATATGCGTAATTACTATGTATAATCCAATCTTTTT

RbSat33246        GAATAAATAAGATATGAATCATATTCTAGAAAGTATAAAGAATTTAAATGTAGATTATTGTGATGAAATGACTTAG-156
                  ||||||||||||||||||||||||||||||||||||||||||||||||||||||||||||||||||||||||||||
Tyba-satDNA       GAATAAATAAGATATGAATCATATTCTAGAAAGTATAAAGAATTTAAATGTAGATTATTGTGATGAAATGACTTAG-172

(B) Ty1-copia-like x gi|294863870|gb|ADF45865.1|:6-87 reverse transcriptase, [Eleocharis ovata]
Score             Expect      Method                  Identities   Positives    Gaps
171 bits(433)     2e-61       Comp. Matrix Adjust.    82/82(100%) 82/82(100%) 0/82(0%)

E. ovata     GNLEEDVYMTQPECFVDPKNANKVCKLQRSIYGLKQASRSWNKRFDDEVKKLNFIQSEKEPCVYKRISGSLIVFLVLYVDDM  87
             GNLEEDVYMTQPECFVDPKNANKVCKLQRSIYGLKQASRSWNKRFDDEVKKLNFIQSEKEPCVYKRISGSLIVFLVLYVDDM
Rb-copia     GNLEEDVYMTQPECFVDPKNANKVCKLQRSIYGLKQASRSWNKRFDDEVKKLNFIQSEKEPCVYKRISGSLIVFLVLYVDDM  82

(C) Ty3-gypsy-like x gi|22296822|gb|AAM94350.1|:644-888 gag-pol polyprotein [Zea mays]
Score             Expect      Method                  Identities         Positives          Gaps
390 bits(1001)    5e-144      Comp. Matrix Adjust.    187/187(100%)      187/187(100%)      0/187(0%)
Z. Mays      1-GLPPIRGIEHQIDLIPGASLPNRAPYRTNPEETKEIQRQVQELLDKGYVRESLSPCAVPVILVPKKDGTWRMCVDCRAIN-80
               GLPPIRGIEHQIDLIPGASLPNRAPYRTNPEETKEIQRQVQELLDKGYVRESLSPCAVPVILVPKKDGTWRMCVDCRAIN
Rb-gypsy     1-GLPPIRGIEHQIDLIPGASLPNRAPYRTNPEETKEIQRQVQELLDKGYVRESLSPCAVPVILVPKKDGTWRMCVDCRAIN-80

Z. Mays        NITIRYRHPIPRLDDMLDELSGAIVFSKVDLRSGYHQIRMKLGDEWKTAFKTKFGLYEWLVMPFGLTNAPSTFMRLMNEV-160
               NITIRYRHPIPRLDDMLDELSGAIVFSKVDLRSGYHQIRMKLGDEWKTAFKTKFGLYEWLVMPFGLTNAPSTFMRLMNEV
Rb-gypsy       NITIRYRHPIPRLDDMLDELSGAIVFSKVDLRSGYHQIRMKLGDEWKTAFKTKFGLYEWLVMPFGLTNAPSTFMRLMNEV-160

Z. Mays        LRAFIGKFVVVYFDDILIYSKSMDEHV-187
               LRAFIGKFVVVYFDDILIYSKSMDEHV
Rb-gypsy       LRAFIGKFVVVYFDDILIYSKSMDEHV-187
```

Figure S3. Sequences and alignment of repetitive DNA families used for *primer* design. (**a**) Rbsat33246, represents a satDNA with a 172 bp repeat similar to the Tyba-satDNA described for the related species *R. pubera*. (**b**) 246 bp long Rb-*copia* probe, encoding an 82 amino acid region with high similarity to the reverse transcriptase of some LTR retrotransposons in multiple grass species. (**c**) 561 bp long Rb-*gypsy* probe, encoding an 187 amino acid region similar to the reverse transcriptase region in several *Zea mays* LTR retrotransposons.

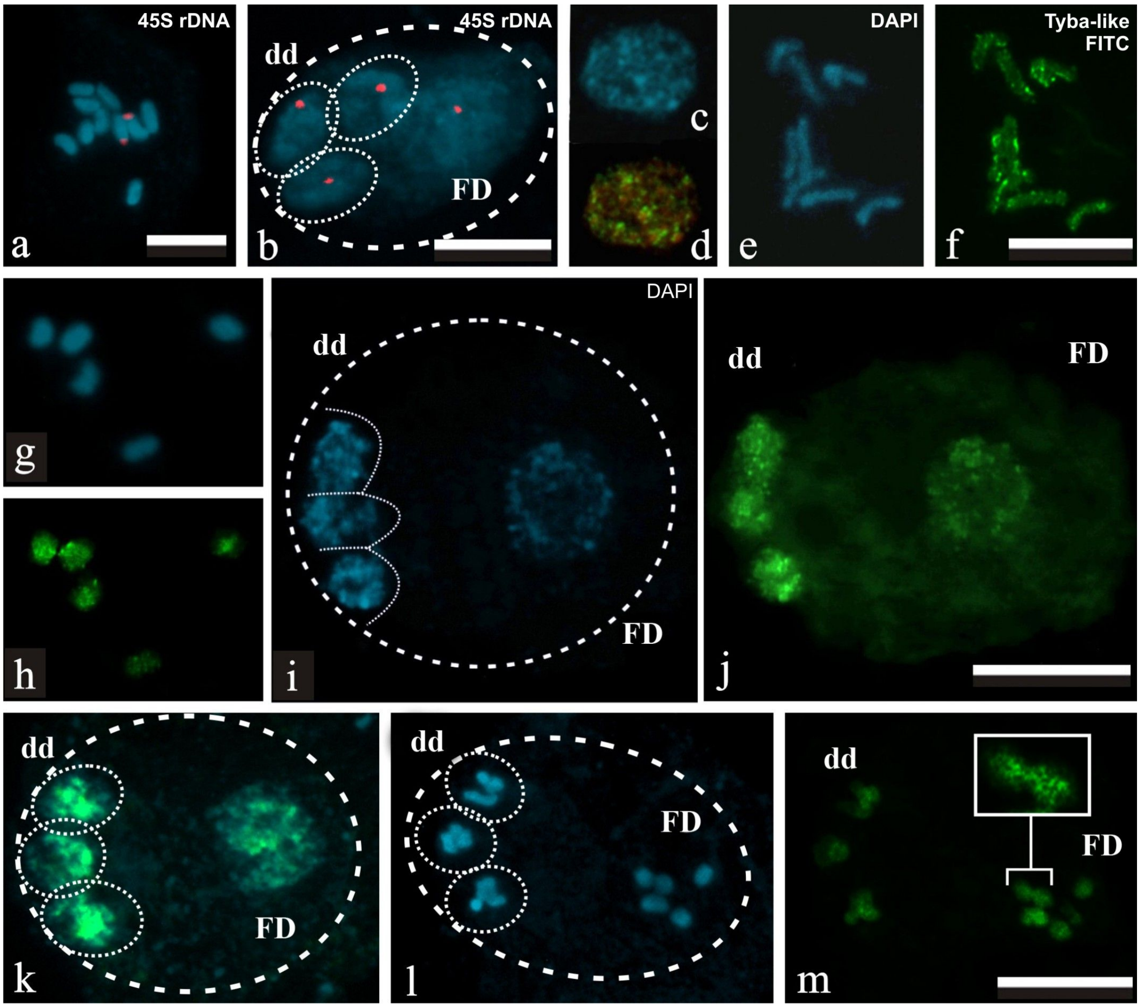

Figure S4. Fluorescent *in situ* hybridization in *R. breviuscula* using 45S rDNA and Rbsat33246 probes. Nucleus is counter-stained with DAPI (blue) or pseudo-colored in red in d. (**a**) FISH using a 45S ribosomal DNA (p*Ta*71 clone) probe labeled with digoxigenin (red). Note two terminal signals in mitotic metaphase (**a**) and one hybridization signal in each of the four nuclei (**b**). Scale bars = 10 µm. FISH using the Tyba-like Rbsat33246 probe. Interphase nucleus from somatic tissues stained with DAPI (**c**) and merged with FISH (**d**). Note the signals (green) spread across its entire nucleus. (**e-f**) Eight of ten prometaphasic chromosomes of a somatic cell can be seen stained with DAPI (**g**) and hybridized with the Rbsat33246 probe (**h**). Note that these signals (green) locate irregularly along the entire holokinetic chromosome. Scale bar in f, also for c-e = 10 µm. (**g-h**) Five bivalents (metaphase I) stained with DAPI (**g**) and hybridized with the Rbsat33246 probe (**h**). The hybridization signals were apparently scattered. (**i**) Pseudomonads of *R. breviuscula* stained with DAPI. Three nuclei with most condensed chromatin can be seen on the left edge of the pseudomonad in the

degenerative domain (dd), while a large decondensed nucleus is present in the functional domain (FD). (**j**) When the Rbsat33246 probe (green) was used for FISH on pseudomonad nuclei, degenerative nuclei exhibit more intense hybridization signals when compared to the functional nucleus, probably due to differential chromatin condensation. Scale bar in j, also for g-i = 10 μm. (**k**) Pseudomonads in PM I stained with DAPI and merged with the Rbsat33246 probe, showing a slightly more advanced stage with degenerative nuclei also more condensed. (**l-m**) FISH using the Rbsat33246 probe in PMI showing that signals have similar hybridization intensity. Note, during this stage, chromosomes in the degenerative domain (dd) are smaller than those in the functional domain (FD), and there is no noticeable difference in the hybridization signal intensity, except the small size of non-replicated chromosomes. Scale bar m, k and l = 10 μm.

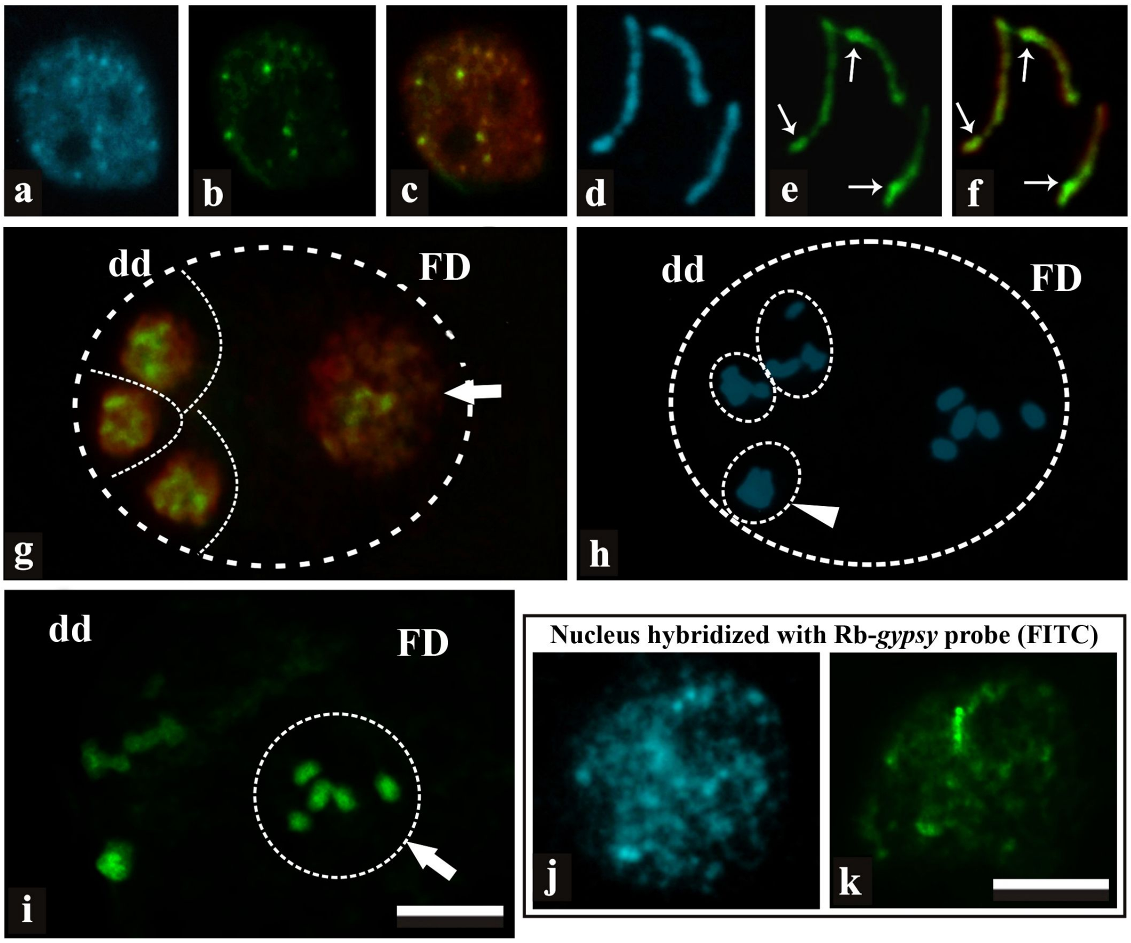

Figure S5. Fluorescent *in situ* hybridization of *R. breviuscula* using Rb-*copia* and Rb-*gypsy* probes. DAPI was pseudo-colored in red in c, f and g. (**a**) Interphase nucleus from somatic tissue stained with DAPI (blue) and (**b**) Rb-*copia* signals (green) accumulated in the chromocenters. Note that FISH signals are located in chromocenter regions (**c**). (**d**) Three chromosomes of a somatic cell can be seen stained with DAPI and their respective FISH signals. (**e**) Note Rb-*copia* signals (green) predominantly localize at the chromosome ends (**e-f**). (**g**) FISH of pseudomonads presents three degenerative nuclei (pseudocolored in red for improving contrasts) exhibiting a condensed chromatin, with intense hybridization signals in this domain (dd), while a large, decondensed functional nucleus (red) with less strong signals can be observed in the functional domain (FD). (**h-i**) FISH with Rb-*copia* in a pseudomonad during PM I shows less bright hybridization signals on degenerative chromosomes in dd, in relation

to chromosomes in the FD (arrow in i). Arrowhead in h indicates five overlapping chromosomes Scale bar in I, also for a-h = 10µm. (**j-k**) FISH the with Rb-*gypsy* probe in interphase nucleus from a somatic cell stained with DAPI (j) and hybridization signals (green) accumulated in different regions of the nucleus, including some chromocenter regions (k). Scale bar in k, also for j = 10µm.

Table S1. Total nuclei count of DNA C-values done in flow cytometry using *R. breviuscula* and *R. pubera.*

Control	Events	Fluo	CV	pg
RP	7522	158462.48	0.05	0.85
RB	11431	38177.67	0.06	3.53

Pseudomonads	Events	Fluo	CV	pg
2C	6680	37495.40	0.05	0.84
C	17102	20645.78	0.03	0.46
D (total)	105353	11377.67	0.26	0.25
D0	10452	16977.16	0.04	0.38
D1	11672	15065.38	0.03	0.34
D2	19384	13060.18	0.05	0.29
D3	20139	10957.86	0.05	0.24
D4	19090	9397.71	0.04	0.21
D5	25877	8062.62	0.05	0.18

RP = *Rhynchospora pubera* diploid nuclei from leaf tissue. RB = *Rhynchospora breviuscula* diploid nuclei from leaf tissue. 2C = Diploid nuclei from *R. breviuscula* pseudomonads. C = Haploid nuclei from *R. breviuscula* pseudomonads. D (total) = measurement of the entire population of degenerative nuclei. D0 – D5 = measurements taken from five different stages of pseudomonads development. Flou = fluorescence values. CV = coefficient of variation. Pg = value in picograms.
